# Optical and mechanical properties of streptavidin-conjugated gold nanospheres through data mining techniques

**DOI:** 10.1038/s41598-020-72534-1

**Published:** 2020-10-01

**Authors:** Simone Peli, Andrea Ronchi, Giada Bianchetti, Francesco Rossella, Claudio Giannetti, Marcella Chiari, Pasqualantonio Pingue, Francesco Banfi, Gabriele Ferrini

**Affiliations:** 1 Interdisciplinary Laboratories for Advanced Materials Physics (I-LAMP), Università Cattolica del Sacro Cuore, 25121 Brescia, Italy; 2 Dipartimento di Matematica e Fisica, Università Cattolica del Sacro Cuore, 25121 Brescia, Italy; 3 Department of Physics and Astronomy, KU Leuven, Celestijnenlaan 200D, 3001 Leuven, Belgium; 4 NEST, Scuola Normale Superiore and CNR – Istituto Nanoscienze, piazza San Silvestro 12, 56127 Pisa, Italy; 5 Istituto di Chimica del Riconoscimento Molecolare, CNR, Milano, Italy; 6 FemtoNanoOptics Group, Université de Lyon, CNRS, Université Claude Bernard Lyon 1, Institut Lumière Matière, F-69622 Villeurbanne, France; 7 Fondazione Policlinico Universitario A. Gemelli IRCSS, Rome, Italy; 8 Dipartimento di Neuroscienze, Università Cattolica del Sacro Cuore, Rome, Italy

**Keywords:** Physics, Nanoscience and technology, Nanoscale materials, Techniques and instrumentation, Nanoscale materials, Techniques and instrumentation, Materials science, Structure of solids and liquids, Microscopy, Optics and photonics, Optical techniques, Near-infrared spectroscopy

## Abstract

The thermo-mechanical properties of streptavidin-conjugated gold nanospheres, adhered to a surface via complex molecular chains, are investigated by two-color infrared asynchronous optical sampling pump-probe spectroscopy. Nanospheres with different surface densities have been deposited and exposed to a plasma treatment to modify their polymer binding chains. The aim is to monitor their optical response in complex chemical environments that may be experienced in, e.g., photothermal therapy or drug delivery applications. By applying unsupervised learning techniques to the spectroscopic traces, we identify their thermo-mechanical response variation. This variation discriminates nanospheres in different chemical environments or different surface densities. Such discrimination is not evident based on a standard analysis of the spectroscopic traces. This kind of analysis is important, given the widespread application of conjugated gold nanospheres in medicine and biology.

## Introduction

Ultrafast optical spectroscopy experiments made substantial contributions to the understanding of the electronic and thermo-mechanical dynamics of solids with nano-metric dimensions^1,2^. Perhaps the most studied examples are metallic nanostructures in the form of thin films, spheres, pillars, cylinders, pyramids, with dimensions of the order of 10 nm, that have generated a conspicuous amount of literature^3–6^. As a non-exhaustive list of investigations on the impulsive thermo-mechanics of paradigmatic systems made of metallic nanostructures in a dielectric environment we mention thin films^7,8^, nanoparticles layers^9–11^, spheres^1^, disks^12–14^ and periodic gratings^15^. The electrons excitation by light pulses and the subsequent energy flow to the lattice with the associated mechanical excitations have been thoroughly investigated, generating several theoretical models (essentially based on the classical two-temperatures model or implementing the Boltzmann equation)^16–18^. Recently the investigations aimed at understanding the thermo-mechanical dynamics of nanostructures inside a host matrix, to take into account the influence of the matrix material and the interfaces^12,19–22^. The time-resolved experiments are usually conducted on well-characterized and possibly single nanostructures, to avoid the averaging process due to the statistical dispersion of dimensions, shapes, and interface properties that occurs working with aggregated nanostructures^2,23–26^. These kinds of experiments can be rationalized with a bottom-up approach, modeling the opto-thermal response starting from a detailed knowledge of the investigated sample.

Here we explore a different scenario, where the system is complex and ill characterized compared to the experiments cited in the preceding paragraphs. Time-resolved measurements performed on these systems are not amenable to bottom-up modeling. We, therefore, implement data mining techniques to classify time-resolved traces and gain physical insight^27^. This approach does not rely on previous knowledge of the investigated system and we, therefore, address it as a top-down approach. For these reasons, as a paradigmatic model system, we investigate functionalized gold nanospheres adhered to an optically transparent surface via complex molecular chains.

We select gold nanoparticles because of their chemical inertness and their low toxicity when used in biological systems. Other metallic nanoparticles do not share the same properties. From an optical point of view, it is known that Cu nanoparticles are prone to surface oxidation that affects their optical properties^28^. Ag nanoparticles are known to be unstable under a process known as oxidative dissolution, consisting of a release of Ag+ ions through an oxidative mechanism. Moreover, Ag nanoparticles are subject to photo-oxidation processes that change their optical properties^29^. Both Ag and Cu nanoparticles are mainly used for their antibacterial properties^30,31^.

For the gold nanospheres system, one of the major issues was the reproducibility of the experimental data. The statistical dispersion of measured parameter was indeed high, due to the low transmission modulation induced by the optical pump on the system, the different chemical environments to which each group of the gold nanospheres under scrutiny was subject, the impossibility of precisely define the measurement conditions. Such problems arise while dealing with samples escaping a thorough characterization or while performing *in vivo* measurements of biological interest^32^, due to the impossibility to gather a deep knowledge of the samples of interest. Since a measurement scenario as the one described above has a great interest in applications and/or technological developments, the fundamental question relates to the possibility of extracting some meaningful information from statistical methods requiring the smallest number of measurements.

**Figure 1:**
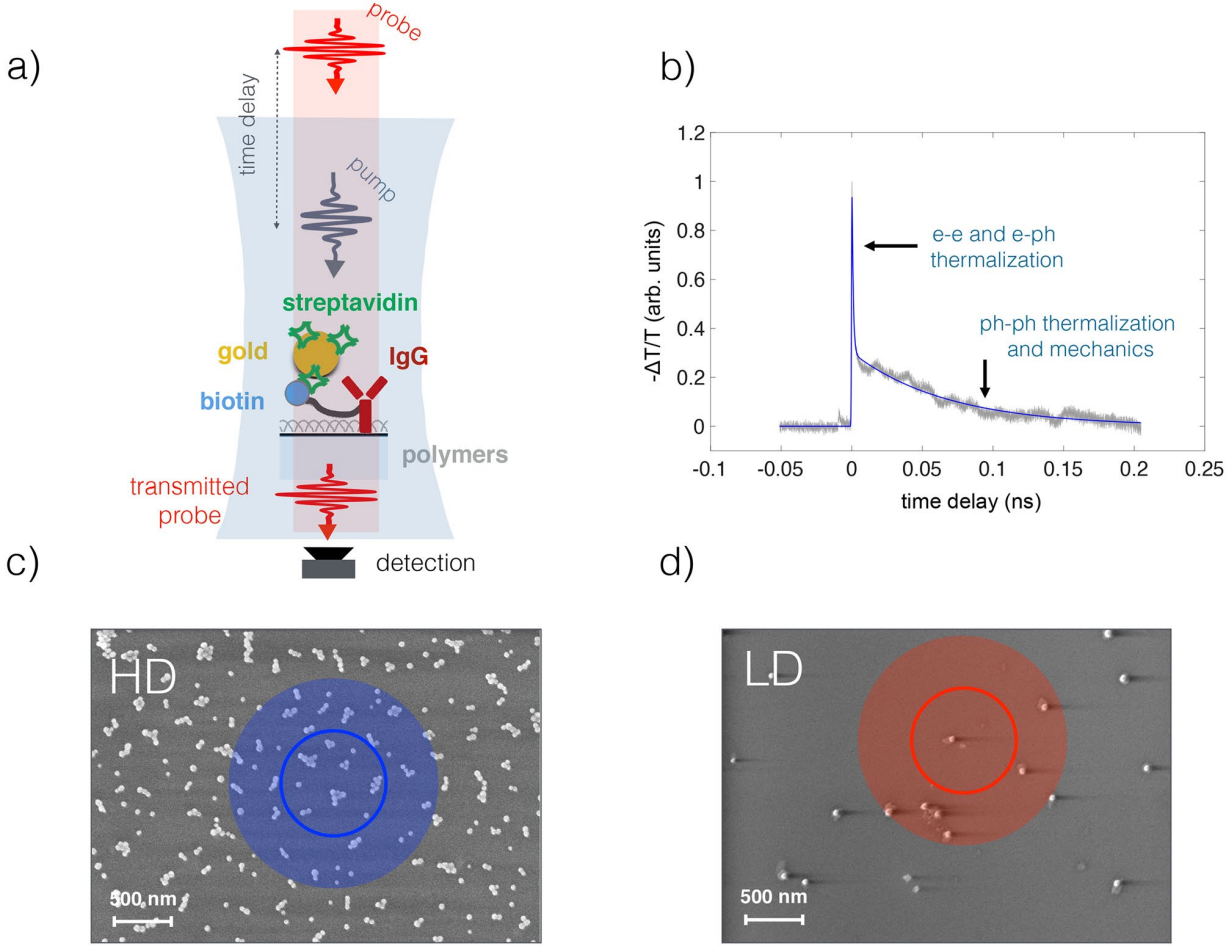
(**a**) A scheme of the pump-probe technique and the composition of the sample. A streptavidin-coated gold nanosphere is attached to a biotinylated antibody which is polymer-tethered to a sapphire surface. (**b**) A representative time-resolved experimental trace, with the indication of the physical processes contributing at different delays, see text for discussion. (**c**) A scanning electron micrography of a high density sample, with an average surface density of 10 clusters, $\upmu \hbox {m}^{-2}$. Each cluster is on average composed of $3 \pm 2$ nanospheres. The shaded blue disk represents the pump spot, the continuous blue circumference represents the probe spot. (**d**) A scanning electron micrography of a low-density sample, with a surface density of 1–2 nanospheres $\upmu \hbox {m}^{-2}$. The shaded red disk represents the pump spot, the continuous red circumference represents the probe spot.

In the present study, the time-resolved experiments (see Fig. 1a,b) are carried out using ASynchronous OPtical Sampling (ASOPS). This choice is tailored to scenarios requiring fast acquisition time, eventually in conjunction with a microscopy set-up. Indeed, ASOPS has proven very successful in mechanical nanometrology^33–35^, and super-resolution imaging of nano-objects^36^. The pump-probe delay is controlled electronically, avoiding moving parts and providing an ideal setting for single nano-objects investigations on extended time-delays. Besides providing superior beam pointing stability (actually that of the laser cavity), the acquisition speed allowed for high rate sampling yielding unprecedented sensitivity in recent experiments, where relative transmissivity variations as low as $10^{-7}$ where achieved^9^. Furthermore, the rapid acquisition of the time-resolved spectra over delay windows of tens of ns with sub-ps time resolution is well suited for investigating degradable and biological samples, such as imaging of cells structures on a single cell via photoacoustics microscopy^37,38^. The ASOSP technique allows avoiding sample exposure to high-intensity radiation, such as in-vivo photoacoustic imaging^39^, and collecting a statistically significant number of spectra where needed^27^. These latter aspects make the ASOPS technique particularly well suited in conjunction with top-down analysis approaches based on data mining techniques, where information has to be retrieved from extended data sets acquired on possibly degradable samples.

Our experiments are taken on three groups of gold nanospheres, differing for surface density and surface treatment. The goal is to identify specific features of each group, digging data from the measurement made on each group, without other external information besides the measurements themselves. The data analysis used in this work has three main goals: to eliminate outliers, reduce data dimensionality, identify the characteristic response of each group.

## Methods

Functionalized gold nanoparticles, with a nominal diameter of 40 nm, are bonded on an optically transparent sapphire substrate according to the following procedure. The sapphire substrate is coated with a polymeric solution of poly (DMA-co-NAS-co-MAPS)^40^. Then a biotinylated antibody, diluted at different concentrations in a phosphate-buffered saline solution, is spotted on the polymer covered substrate using an automated dispensing system. The spotted substrates are finally incubated with a solution of gold nanospheres conjugated with streptavidin at different concentrations. The high-density samples are obtained using a 1 mg/ml concentration of antibody incubated with and undiluted nanosphere solution. The low sample is obtained with a 0.1 mg/ml concentration of antibody and a nanoparticle solution diluted 1:10. The high-density samples have been successively subject to a plasma treatment to partially remove the polymer coating and expose the bare nanospheres (see the Supplementary Information (SI), section 1, for more details). Scanning electron microscopy and atomic force microscopy have been used to characterize the nanospheres’ surface density and state of aggregation after the measurements. The undiluted sample has aggregated clusters of 1-5 nanospheres, with an average surface density of 10 clusters $\upmu \hbox {m}^{-2}$, see Fig. 1c. The diluted sample is composed mainly of single nanospheres with an average surface density of $1\, \upmu \hbox {m}^{-2}$, see Fig. 1d. See SI, sections 2 and 3 for more details.

There are thus two distinct density groups that we will refer to as the high density (HD) and low density (LD) groups. Among the HD groups, there are two subgroups: HDnp, which did not undergo the plasma treatment, and HDp, representing the plasma-treated nanospheres.

The optical experiments are carried out using the pump and probe technique, see Fig. 1a. The pump pulses are generated by an Er:doped fiber laser at a wavelength of $\lambda _{pu}=1560$ nm. A separate fiber laser generates the probe pulses at a wavelength of $\lambda _{pr}= 780$ nm. Both laser have an average repetition rate of 100 MHz and their cavities are locked to emit two beams that have a small, stable, and tunable repetition rate difference. The laser beams are colinearly directed in a 50$\times$ microscope objective (numerical aperture: 0.55), producing on the sample a $1\, \upmu \hbox {m}$ spot diameter at $\lambda _{pu}=1560$ nm and $0.6 \,\upmu \hbox {m}$ at $\lambda _{pr}=780$ nm. The pump beam has a power of 2 mW (fluence $640 \,\upmu \hbox {J/cm}^2$ on the sample), the probe beam of 0.25 mW (fluence $220 \,\upmu \hbox {J/cm}^2$ on the sample). The transmitted probe pulses are sampled after interacting with the sample, which is optically pumped with the beam coming from the pump laser. The transmission variation is defined as $\Delta T/T= (I^{probe}_{pump, \tau } - I^{probe}_{no pump}) / I^{probe}_{no pump}$, where $I^{probe}_{no pump}$ is the transmitted probe intensity in the absence of the pump pulse, $I^{probe}_{pump, \tau }$ is the transmitted probe intensity at delay $\tau$ after the pump pulse. With this convention, negative transmission variation indicates that pump excitation lowers probe transmission, see Fig. 1b. This ASynchronous Optical Sampling (ASOPS) allows high-speed scanning over a delay window of 10 ns with 100 fs resolution, without a mechanical delay line^41^. This technique routinely measures relative reflectivity or transmission variation as small as $10^{-6}$. The absolute variation in transmission caused by the pump beam is of the order of $\Delta T/T = -5/-2 \times 10^{-5}$. The transmission variation is negative for all measurements.

In the experiments on the LD sample, we expect to have, on average, one-two nanospheres in the probe spot. The HD sample shows, on average, around ten clusters inside the probe spot, composed of few (2-5) particles each. In both cases, the nominal diameter of the nanospheres is about 20 times smaller than the probe wavelength, and their positions on the sample surface are not known *a priori*.

The samples were raster-scanned with the collinear beams until a signal was detected in the probe beam as a relative transmission variation ($\Delta T/T$) induced by the pump. When a signal was detected, its amplitude was optimized with small adjustments around that specific position and a measurement acquired without moving the sample. Since the polymer layer was optically transparent, the presence of the signal was attributed to the presence of the gold nanospheres, modulating the probe transmission via the pump excitation, as is well documented in the literature^1,17^. The measurements were acquired in 12 sessions within two months. The quality of the signal varied from session to session, and scan to scan. However, the data were not discarded based on the experimenter judgment (as usual in these kinds of measurements) but accumulated, to be assessed after all the experimental sessions were completed. A set of 36 time-resolved pump-probe measurements has been performed, 13 measurements on HDnp, 17 on LD, and 6 on HDp.

Before the analysis, the transient transmission data have been normalized at the maximum amplitude. The delay axis has been centered, so that delay zero corresponds to the arrival of the pump pulse on the sample. The normalization aims at disentangling the successive analysis from the magnitude of the pump-induced transient transmission variations, which depends on experimental parameters that may vary from one experimental session to the other as, e.g., centering of nanostructures on the pump beam focus, pump-probe beam alignment, pump intensity fluctuations, etc.

**Figure 2:**
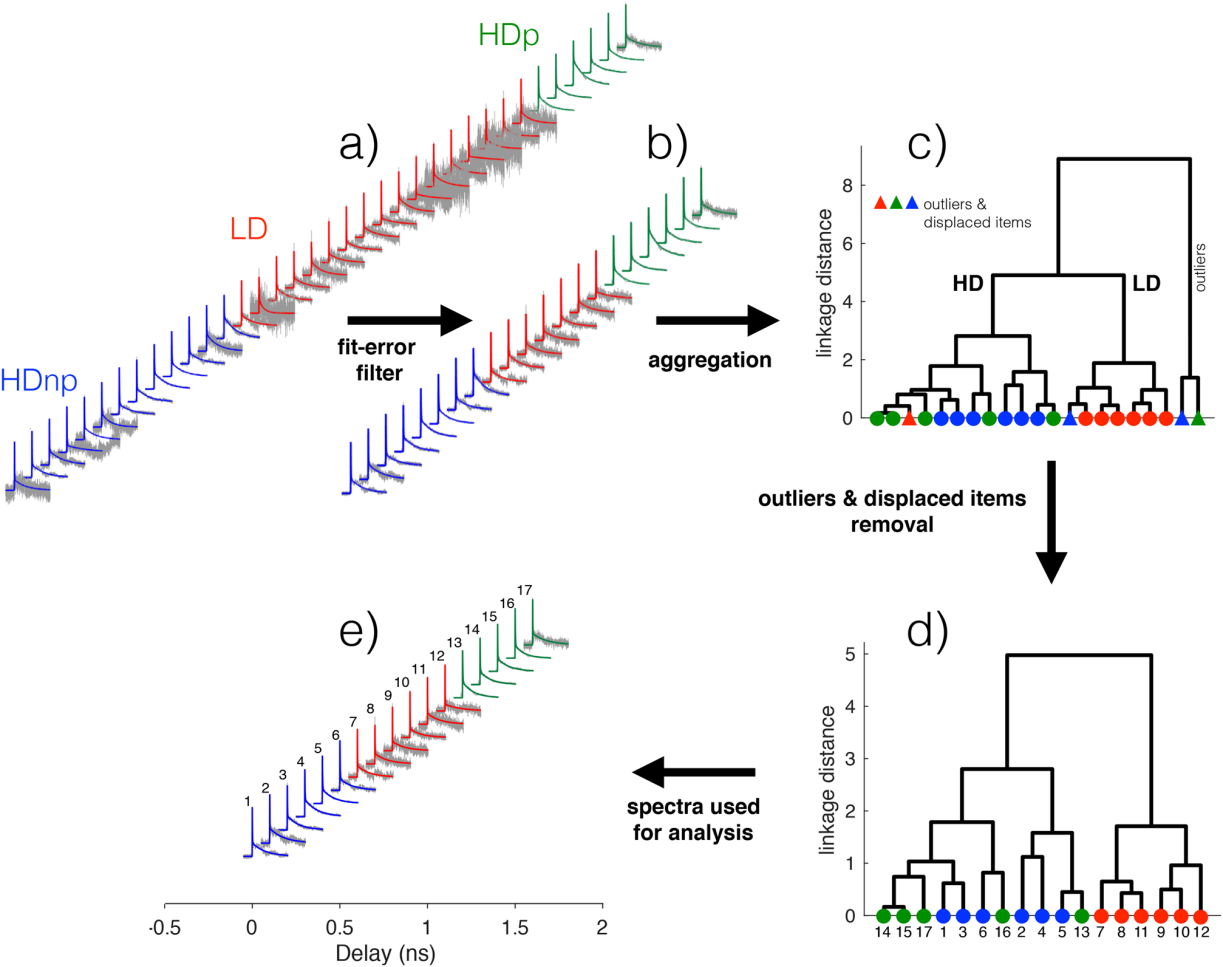
The set of measurements (gray lines, displaced for clarity) and the superposed fit functions (colored continuous lines) are shown in (**a**). The color code is as follows: blue, high density without plasma treatment (HDnp); red, low density (LD); green, high density with plasma treatment (HDp). In (**b**) are shown the filtered spectra using the fitting error as a parameter (see text for details). The results of the hierarchical clustering algorithm based on Ward’s method are shown as a dendrogram in (**c**). The leaves (that constitute the lower terminal part of the dendrogram) represent the spectra as seen in (**b**), ordered hierarchically. The same color code as in (**a**) is used. The dendrogram, pruned away from outliers and displaced items, is shown in (**d**). The remaining spectra, used for subsequent analysis, are shown in (**e**).

**Figure 3:**
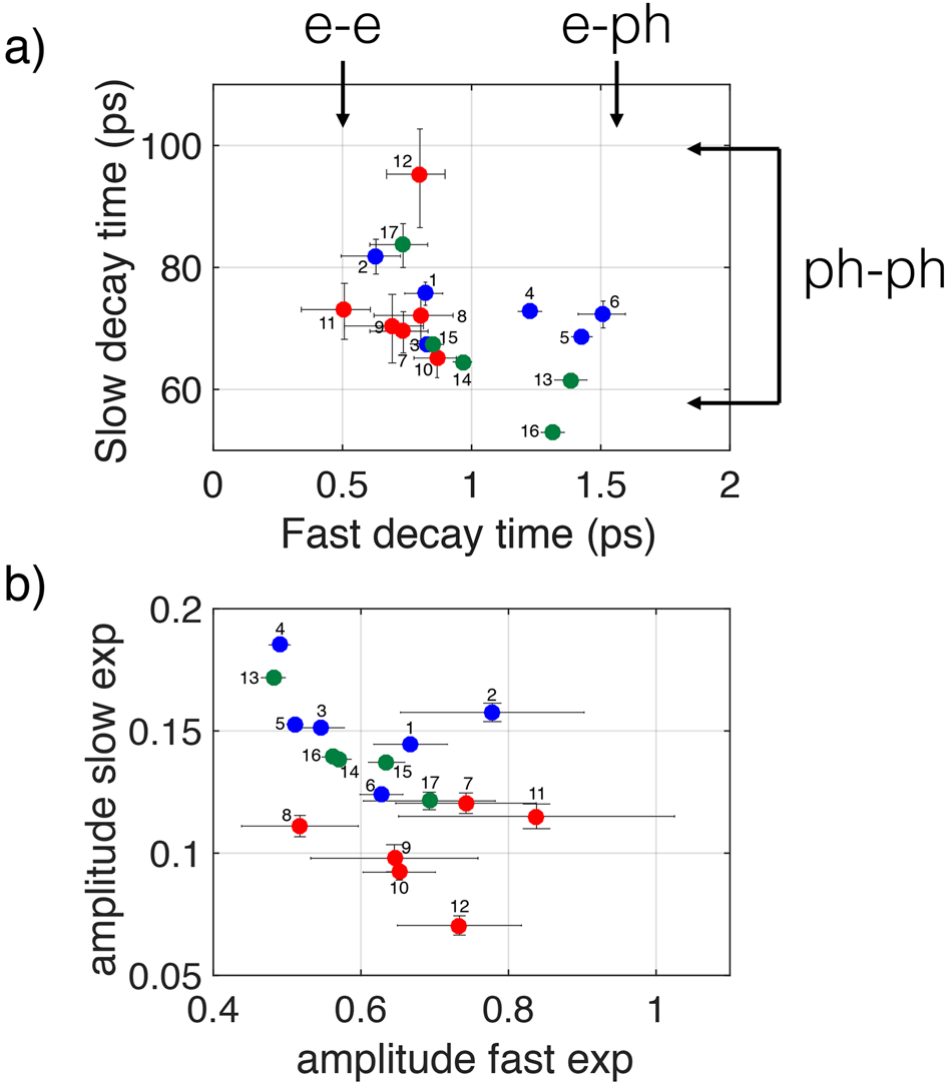
Fitting parameters for the double exponential fit to the data of the selected spectra shown in Fig. 2e. In (**a**) the fast exponential decay times versus the slow exponential decay times are reported. Note that the fast decay times are longer than the electron–electron scattering times (e–e) and slower than the maximum electron–phonon scattering time (e–ph) reported in the literature^3,53,54^. The slow decay time values fall between the limits reported in the literature for phonon–phonon scattering times (ph–ph). In (**b**) the amplitudes of the fast decaying exponentials versus the amplitudes of the slow decaying exponentials are shown.

**Figure 4:**
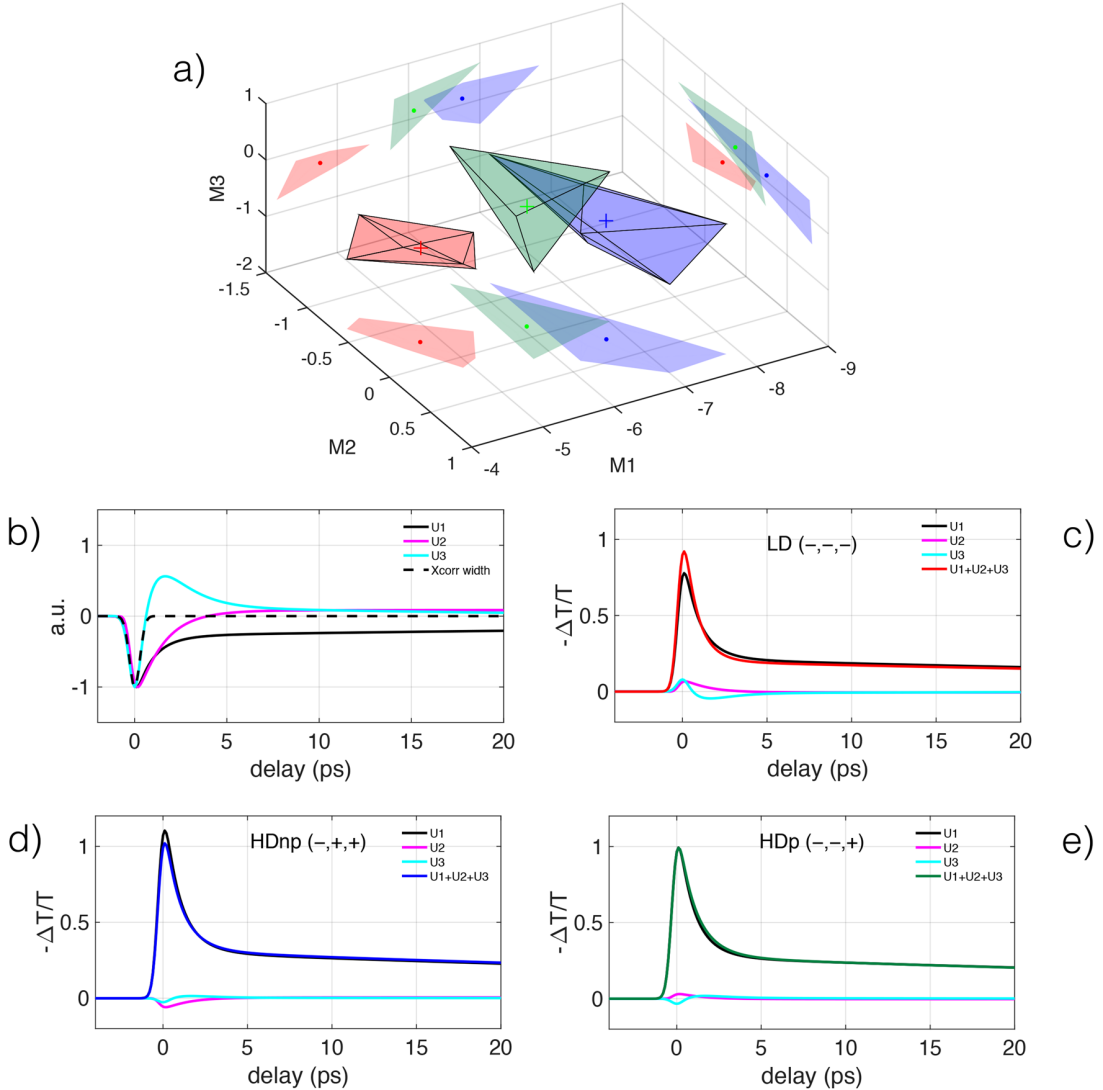
Results from Singular Value decomposition analysis on the data fit functions. In (**a**) are shown the convex hulls of each data group. The convex hulls are shown as the smallest (semitransparent) convex polyhedra that contain all the points of each group. The coordinates (M1, M2, M3) represent the projections of each spectrum onto the three eigenvectors with the highest eigenvalues. The color code is the same as in Fig. 2. In (**b**) the shapes of the first three eigenvectors ($U_1, U_2, U_3$) are shown. The shape of the pump-probe cross correlation is shown as a dashed black line. In (**c**) is shown the reconstructed barycentric trace for the low-density case (red cross in **a**)), obtained by the sum of the first three weighted eigenvectors. The three weighted eigenvectors are shown for comparison with the reconstructed barycentric trace. (**d**) and (**e**), same as c) for the cases of high density without plasma treatment and high density with plasma treatment, respectively.

Each measurement has a temporal evolution starting with a (negative) peak at zero delay due to electron absorption followed by a multiple exponential decays. The temporal evolution is fitted with a double decaying exponential function, convoluted with a gaussian function to take into account the finite temporal resolution of the laser pulse. The fit function is $F_1=\text {exp}(t^2/\tau _{G}^2) *(A_F e^{(t/\tau _F)}+A_S e^{(t/\tau _S)})$, where $\tau _F$ and $\tau _S$ are the fast and slow exponential decay times, respectively, $A_F$ and $A_S$ are amplitudes associated to the fast and slow decaying exponentials, $\tau _G$ is the time-width at 1/*e* of the pulse Gaussian envelope, $*$ is the convolution operator. In Fig. 2, the time-resolved traces with the superposed bi-exponential fits and the filtering method (that will be discussed in the next section) are shown. The fit parameters and the associated errors retrieved from the fit process are reported in Fig. 3. Note that the data have different degrees of noise, depending on the group to which they belong. Diluted measurements are more difficult to perform because of lower signal levels and lower signal-to-noise ratio. This is reflected in the fit function errors, shown in Fig. 3, where the LD group is characterized by wider error bars. Figure 4 shows the analysis of the transient transmission data using the singular value decomposition (SVD), a general data mining technique used for data dimensionality reduction, data visualization, and information analysis^42,43^.

**Figure 5:**
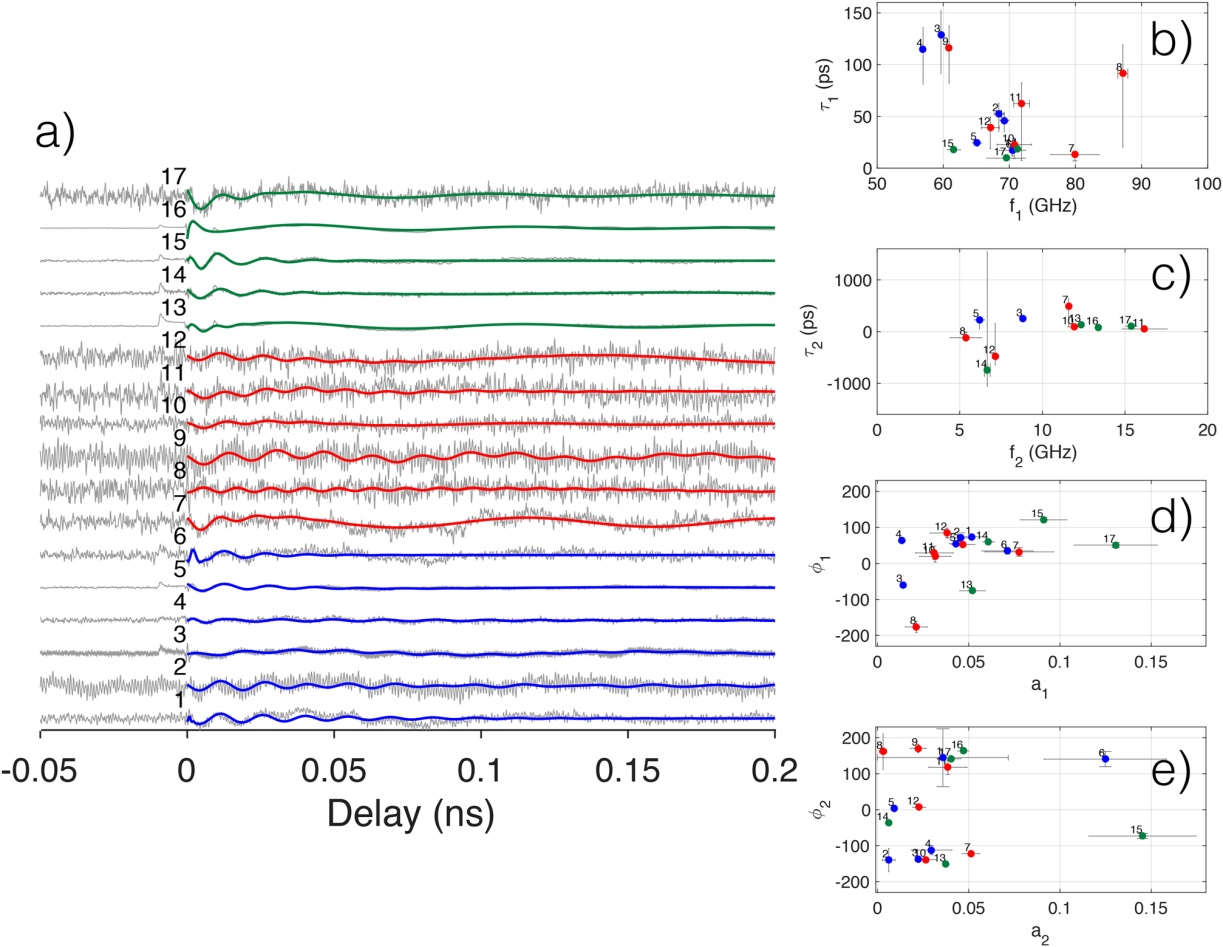
The set of residuals (gray lines, displaced for clarity) and the superposed fit functions (colored continuous lines), are shown in (**a**). In (**b**, **c**) are reported the oscillation frequencies versus associated exponential damping times for the first ($f_1$ vs $\tau _1$) and second ($f_2$ vs $\tau _2$) oscillator. (**d**, **e**) reports the amplitude versus phase for the first ($a_1$ vs $\phi _1$) and the second ($a_2$ vs $\phi _2$) oscillator.

**Figure 6:**
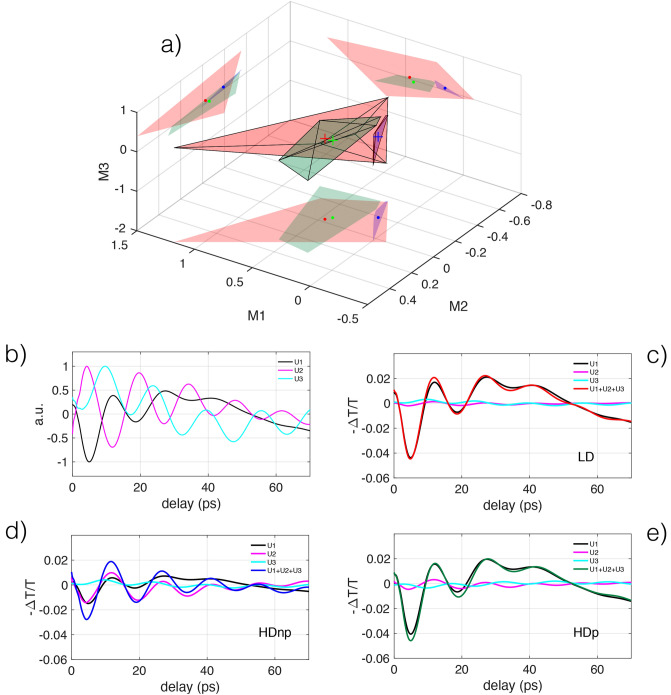
Results from singular value decomposition analysis of the fit functions of the residuals. In (**a**) are shown the convex hulls of each data group. The color code is the same as in Fig. 2. In (**b**) the shapes of the first three eigenvectors ($U_1, U_2, U_3$) are shown. In (**c**) is shown the reconstructed barycentric trace for the low-density case (red cross in (**a**)), obtained by the sum of the first three weighted eigenvectors. The three weighted eigenvectors are shown for comparison with the reconstructed barycentric trace. (**d**) and (**e**), same as (**c**) for the cases of high density without plasma treatment and high density with plasma treatment, respectively.

The analysis of the residuals of the double-exponential fits shows the presence of a periodic modulation, that has been fitted with a sum of damped cosines at two frequencies. The fit function is $F_2=a_1 \text {cos}(2 \pi f_1 t+\phi _1) \text {exp}(-b_1 t)+a_2 \text {cos}(2 \pi f_2 t+\phi _2) \text {exp}(-b_2 t)$, where $a_1$ and $a_2$ are amplitudes, $f_1$ and $f_2$ frequencies, $\phi _1$ and $\phi _2$ phases, $b_1$ and $b_2$ damping rates. $\tau_1=1/b_1$ and $\tau_2=1/b_2$ are damping times associated to the damping rates. In Fig. 5 the residual traces with the superposed damped cosines fit, the fit parameters and the associated errors are shown. Figure 6 shows the analysis of the residuals by SVD, as done for the transient transmission data.

In the next sections, these measurements and their analysis will be discussed.

## Results and discussion

Although the detailed description of light absorption and successive electronic and lattice dynamics is not the focus of the present work, we give a brief description of the main points of the thermal and mechanical dynamics.

The plasmon resonance for gold nanospheres of 40 nm diameter is approximately located at $\lambda = 530$ nm ($h \nu =2.34$ eV), the inter-band transition starts approximately at $h \nu =2$ eV ($\lambda = 620$ nm). So the infrared pump used in these experiments ($\lambda _{pu} = 1560$ nm) is non-resonant and generates a hot electrons distribution in the nanoparticle via intra-band transitions^44^. The hot electrons thermalize in few hundreds of fs and release energy to the lattice in 1–2 ps^3^. The higher nanoparticle temperature, besides changing the optical constants of gold, will determine a temperature gradient directed from the nanoparticle towards the surrounding molecules (polymers chains, biotin, etc.). As a result, an energy transfer takes place along the temperature gradient and the temperature of the molecules will raise, changing the local refraction index^12,21^. Also, the raising lattice temperature will cause an impulsive expansion of the nano-sphere diameter^1,45,46^. Since spherical particles are endowed with normal oscillation modes (oscillation eigenmodes), the mechanical expansion consists of the linear combination of few lower normal modes, called breathing modes^1^. It is found experimentally that modulation of the probe transmission is present at the frequency of the breathing mode for 40 nm diameter gold spheres. The standard mechanism to rationalize probe modulation in time-resolved experiments, described in the literature, is based on the periodic shift of plasmon resonance, which depends on the nanoparticle volume^1^. In our case, the pump is very far from resonance, so it is not obvious if the plasmon modulation provides the dominant contribution to the observed probe modulation. Other candidates could be the pump-induced modulation of the dielectric constant of gold or the nanospheres’ mechanical modulation of their molecular corona. More details on the absorption cross-section, the electron and lattice temperatures, and the pump-induced variation of the cross-section in SI, sections 4, 5, 6.

In any case, we are seeking dissimilarities between nanospheres probed in different conditions irrespective of the precise physical mechanism of excitation. The analysis is divided into two parts. First, the probe transmission variation induced by the pump is studied, without taking into account eventual rapid modulations of the signal, by fitting the initial peaked response with decaying exponentials. Then the residuals are fitted with damped oscillating cosines to extract the periodic signal modulation.

### Thermal analysis

Each measurement has been fitted by two-component decaying exponential functions to capture the fast electronic response and the slower thermal dynamics, a well-documented procedure in the literature^19,27,47^. The quality of fit is generally good, see Fig. 2a, however several measurements have a high level of noise and/or varying shape that we ascribe to non-optimal measurement conditions. To obtain a set of data with an overall good quality, we filter data based on the error of the fitting parameters, keeping the measurements whose decay times are subject to an error that is less than 25% of the decay times values. We note that the high errors on fit parameters we found in some traces are not due to a failure of the fitting model, but to increased noise, as confirmed by the residual analysis. After this selection, 21 measurements (8 HDnp, 7 LD, 6 HDp ) are considered for further analysis, out of the initial 36, Fig. 2b. We will refer to these measurements as *filtered measurements*. To assess the general consistency of the data and isolate eventual outliers, data were aggregated using a hierarchical cluster analysis, known as Ward’s minimum variance method^48,49^. It does not require to pre-specify the number of clusters to be generated. The method agglomerates data adding items one at a time, associating each item to a group with the criterion to minimize the total within-cluster variance. In our case, a Euclidean metric is used to calculate the distance between experimental traces. See SI, section 7, for a detailed description of the method.

Outliers are commonly seen as “cases that do not follow the same model as the rest of the data”^50^. In this broad definition are included items known to belong to a class and classified in another. We will refer to these items as *displaced items*^51,52^. Our strategy is to use hierarchical cluster analysis as a guide to obtaining distinct groups, guided by the known characteristics of the samples, i.e., density variation and eventually plasma treatment. We follow these steps: (a) aggregate data and remove outliers, if any; (b) aggregate data without the outliers and remove the displaced items to obtain distinct groups, if possible; (c) use the remaining items for further analysis. Step (b) is crucial, and it is possible only if the data are of sufficiently high quality to have a low number of outliers and if data show a neat clustering tendency, with a small number of displaced items. In this way, we purify the spectral characteristics belonging to a specific group by eliminating displaced items from that group, that we attribute to a statistical and unpredictable variation in the measured data, as explained above.

The result of the hierarchical cluster algorithm is a dendrogram that visually represents the distances between items and identifies clusters of items. The analysis of the 21 spectra remaining after the filtering gives a dendrogram whose root node divides the measurements into two groups, one where the majority of the measurements are found in the fundamental cluster and another with only two elements, so distant from all the others to be classified as outliers and excluded from subsequent analysis. The fundamental cluster divides the measurement into two sub-clusters (or leaves), one with a prevalence of HD measurements and the other with a prevalence of LD measurements, Fig. 2c. The shape of the two leaves shows that the one containing mainly LD traces (red) has a reduced distance between items compared to the other leaf, containing mainly HD traces, with a more fragmented aspect. This suggests that traces in the LD group have less inter-group distance and consequently are more “similar” among themselves than those in the HD group. It is important to note that the notion of similarity here depends on the metric used to calculate the traces distances.

The displaced items in the two groups are eliminated (one item in the HD group, one item in the LD group). We will refer to this analysis as *outliers removal*. After outliers removal, 17 spectra remains (6 HDnp, 6 LD, 5 HDp), their dendrogram is shown in Fig. 2d. The silhouette criterion confirms that the optimal number of clusters for this distribution is 2^49^. The remaining spectra with their fitting functions are shown in Fig. 2e. For reference, the selected spectra have been numbered.

Is the clustering shown in Fig. 2d) evident from the distribution of the fit parameters? In Fig. 3a) we show the fast decay time plotted against the slow decay time. The three groups do not show any significant formation of separate clusters, the point of all group being intermixed. Same conclusions if we plot the amplitude coefficients of the fast decay exponentials versus the amplitude coefficients of the slow decay exponentials, Fig. 3b. We reach the same conclusions by plotting the fitting parameters in other combinations. We note that the fast and slow decay times have values within bounds found in the literature: electron-electron thermalization and electron-phonon decay time are expected to range from 500 fs to 1.5 ps^3,53,54^, while the slower phonon-phonon coupling of the particle to its surrounding medium implies a decay time of the order of 80-100 ps in gold nanoparticles^3,53^.

Now we analyze the selected data using the singular value decomposition (SVD)^42,43^. SVD can be applied to any $m\times n$ matrix *X* and provides a factorization into the product of three matrices, $X=USV^T$, susceptible of an interesting interpretation. *U* is an $m\times m$ (real or complex) unitary matrix, *V* is an $n\times n$ (real or complex) unitary matrix, S is a $m\times n$ non-negative, real, diagonal, rectangular matrix. The columns of *U* and *V* form a set of orthonormal vectors, called left-singular eigenvectors and right-singular eigenvectors respectively, the associated eigenvalues in *S* are ordered in descending magnitude along the diagonal.

The SVD decomposition has an important approximation property. To approximate a matrix *X* with another matrix ${\hat{X}}$ of lower rank *r*, the solution is given by the SVD of *X* as ${\hat{X}}=U{\hat{S}}V^T$, where ${\hat{S}}$ is the diagonal matrix *S* containing only the *r* highest magnitude eigenvalues and replacing the others by zero^42,43^. This allows us to approximate *all* the column vectors in the matrix *X* using a truncated version of *U*, i.e. using a maximum of *r* eigenvectors. The $n\times n$ matrix $M=SV^T$ contains the coefficients to reconstruct the original data using the left-singular vectors as a base. Using only the first three eigenvectors for the reconstruction, it is possible to obtain an approximate reconstruction and reduce considerably the dimension of the data space. It is possible to demonstrate that, for a given dimension, this approximate reconstruction is the best obtainable in the least square sense.

It is important to underline the problem of normalization of data. It is well known that hierarchical analysis and SVD depend critically on data normalization. However, there is no consensus on which kind of normalization is considered best practice. In our case, we have normalized the experimental traces to unity at the highest value of the transient transmission and have used the best fits to the normalized traces as the input to the statistical analysis, without further pre-processing. As a consequence, the processed data are not centered, as is usually done in this kind of analysis. The idea behind analyzing the fitting functions, not directly the data, is that the shape of the fitting functions is sufficiently rich in information to disentangle the characteristics features of each group while avoiding the masking effect of noise.

We project each of the 17 fit functions on the three eigenvectors, to retrieve the projection coefficients that characterize uniquely each single measurement in this reduced data space. The projection coefficients $M^i_1, M^i_2, M^i_3$ are the weights relative to the $i_{th}$ experimental trace, that is approximated by a weighted sum of the three eigenvectors ($U_1, U_2, U_3$) as $E^i=M^i_1\times U_1+M^i_2\times U_2+M^i_3\times U_3$. The projection coefficients $M^i_1, M^i_2, M^i_3$ can be represented in a three-dimensional *projection space*. Instead of representing one point for each measurement in the projection space, we visualize the points distribution via the convex hulls for each data group. The convex hull is the smallest convex polyhedron that contains all the points of each group. In Fig. 4a the convex hulls of each data group are shown as colored semitransparent polyhedra. The barycenter of each group is shown with a cross. The barycenter position is also projected on the 2D representations on the limiting planes of the 3D graph. The polyhedron containing the LD group (red), is distinct from the other two, containing the HD samples. The SVD analysis separates the data based on a different nano-spheres surface density. The polyhedra containing the HDnp (blue) and HDp (green) are partially superposed and have well-separated barycenters. This shows that the plasma treatment affected the optical properties of the HD nano-spheres to a minor degree compared to density variation.

The normalized shape of the three eigenvectors is shown in Fig. 4b. The eigenvectors’ shape can be correlated to the evolution of the electronic and thermal degrees of freedom of the gold nanospheres. The first eigenvector represents the measurement backbone, i.e., the dynamic that is most representative of all the experimental data. It appears as a peak due to electron-phonon relaxation within the nanoparticle, with a time width of approximately 1.5 ps, followed by an exponential decay lasting several tens of ps, due to the phonon-phonon thermal dynamics. The second eigenvalue has a peak whose temporal width is comparable to that of the first eigenvector followed, by a zero-crossing and a long exponential tail. The third eigenvalue is characterized by a peak with a time width much shorter than the typical electron-lattice relaxation time that is identical to the time resolution of the experiment, given approximately by the Gaussian FWHM width of 600 fs, representing the pump-probe cross-correlation. As we will see, the second and third eigenvalues have a much smaller contribution to reconstructing data and can be interpreted as showing the *differences* between experiments. Differences between measurements due to fast, out-of-equilibrium electronic processes are captured by the third eigenvector.

The barycentric trace, reconstructed using the barycentric coordinates of a specific group (see Fig. 4c–e), represents the characteristic shape of that group and may contain physical information not accessible using an analysis based on the fitting of the data with a model function. To synthesize the barycentric traces we consider the first three eigenvectors ($U_1, U_2, U_3$), shown in Fig. 4b as normalized curves. The crosses in Fig. 4a mark the barycentric coordinated of the convex hulls of each data group. The location of each cross is given by three coordinates $M^b_1, M^b_2, M^b_3$, where *b* is a label to identify the three groups LD, HDnp, HDp. Each M-coordinate represent the weight (projection) of each eigenvector in reconstructing the barycentric traces, as follows ${\mathbb {T}}^b=M^b_1\times U_1+M^b_2\times U_2+M^b_3\times U_3$ where ${\mathbb {T}}^b$ is the barycentric trace of the *b*-th group. The barycentric trace ${\mathbb {T}}^b$ and the three weighted curves that synthesize each ${\mathbb {T}}^b$ (i.e. $M^b_1\times U_1, M^b_2\times U_2, M^b_3\times U_3$) are shown in Fig. 4c–e. It is interesting to study the sign of the projection coefficients needed to reconstruct the average spectrum. By denoting the projection coefficient signs relating to the three eigenvectors with the notation (sign(U1), sign(U2), sign(U3)), we have the following results: LD $(-, -, -)$, Fig. 4c; HDnp $(-, +, +)$, Fig. 4d; HDp $(-, -, +)$, Fig. 4e. Each group is identified by a different sign combination. The magnitude of the first eigenvector $U_1$ is by far the largest, has the same (negative) sign for all cases. $U_1$ determines the average shape of the trace for the three groups (in the figure is reported the negative of the transient transmission). The second and third projections ($U_2$ and $U_3$), considered as corrections to the main eigenvector, show a characteristic pattern for each group. In the cases of LD and HDnp groups, $U_2$ and $U_3$ have the same sign and peak near zero-delay, tending to sum up and modifying the shape of the main peak of the first eigenvector by increasing (LD) or decreasing (HDnp) the transmission compared to $U_1$. Instead, in the HDp group, the peaks of $U_2$ and $U_3$ tend to cancel out and, as a result, only minor modifications are brought to the shape of $U_1$. The transient transmission peak decay in the LD group is faster compared to the average trace (first eigenvector, $U_1$), while the transient transmission in the HDnp group is longer. The increase in transmission in the LD group modifies the transient transmission trace, with an ultrafast component that steepens the leading edge that is absent in the HDnp group. Our data analysis highlights the influence of adsorbates on the gold nanospheres electron-phonon dynamics, that is already described in the literature in some specific cases^55^.

### Mechanical analysis

In Fig. 5a are shown the residuals of the double exponential fits (gray lines) and the superposed double damped-cosines fits (continuous colored lines). In Fig. 5b–e are shown the fit parameters with the error bars. Not all the fits to the residuals of the spectra analyzed in the thermal analysis section give reasonable errors on fit parameters. Selecting measurements for thermal analysis do not guarantee the quality of the measurements on mechanical oscillations. In Fig. 5b–e we zoom in a range of values of the fitting parameters where points are found to group together. As a result only a subset of the fitting parameters referring to the initially selected spectra in Fig. 2 are shown. Note that the number identifying a single measurement may not be present in all the graphs. The graphs showing the complete set of fitting parameters are reported in the SI, section 8. The subsequent SVD analysis is made using all the fits, independently on their quality. This choice is determined by the fact that the fitting parameters of a single trace may have very different error levels. Since excluding a fit because of a larger error in one fitting parameter (but not in the others) involve a degree of arbitrariness, we prefer to keep in the subsequent analysis all the traces. It will be evident that the SVD analysis will give reasonable results, being very robust with respect to data quality.

There are two oscillation intervals where most of the analyzed nanoparticles reside, the higher in frequency around 60–75 GHz, $f_1$ axis in Fig. 5b, the lower around 5–15 GHz, $f_2$ axis in Fig. 5c. The higher frequencies interval corresponds to the breathing modes of spherical gold particles spanning a diameter of 35-45 nm. This is in agreement with the nominal particle diameter of 40 nm, taking into account the possible dispersion of the nano-spheres diameter. The damping times associated with the higher frequencies are of the order of few oscillation periods: an average damping time of 50 ps for an average period of 14.3 ps, i.e. roughly 3.5 oscillation periods resulting in an oscillator quality factor $\hbox {Q}=4.5$ (see Fig. 5b).

The lower frequencies have the same order of magnitude associated with the oscillation found in densely polymer-tethered colloidal particles and attributed to polymer chains oscillations^56^. In this case, the damping times are not well defined, being spread around zero (in the interval (− 500, + 500) ps) with some particles having negative damping values, Fig. 5c. This is due to two main reasons: the first is the extremely low signal amplitude (relative transmission variation in the range of $10^{-7}$), the second is that the periods of oscillation (in the range 100–200 ps) are of the same order of the damping times, with an oscillator quality factor $\hbox {Q}\sim 1$.

The amplitudes associated with high frequency oscillations (Fig. 5d), $a_1$ axis) are well defined and of the order of $a_1\sim 0.01-0.1$. Taking into account that the peak relative transmission variation in the exponentials in Fig. 2e is $\Delta T/T = 2-5 \times 10^{-5}$, the present peak relative transmission variation is of the order $\Delta T/T = 2-10 \times 10^{-7}$. The associated phases concentrate in an interval around 90 deg (associated with a sine type oscillation), with a big dispersion that witnesses the varied conditions of the nanoparticles.

The oscillations amplitudes associated with the low-frequency oscillations $a_2$ (Fig. 5e) are even smaller than $a_1$ and many amplitudes are nearly zero. The phases associated with $a_2$ are evenly distributed at all angles.

The SVD analysis of the fits to the mechanical oscillations are shown in Fig. 6. Figure 6a shows the convex hulls associated with the three groups in the projection space. As already noted, all the points associated with the spectra of a group are contained in the corresponding convex hull, represented as a colored semitransparent polyhedron. The polyhedron containing the LD spectra (red) is the most spread out, containing the polyhedra associated with HDnp (blue) and HDp (green). The spread-out of the acoustic response of the LD spectra is accompanied by their higher error bars in the decaying exponential fits in Fig. 3. The HDnp group has the smallest volume, suggesting that the spectra in this group are similar and have low dispersion. This means that the low-density spectra have varied acoustic responses, to be contrasted with the HDnp group, that have characteristic, less varied acoustic oscillations. The plasma action on the high-density group (HDp, green) causes an expansion of the volume in the projection space, making the acoustic response more varied, more similar to the LD case. The above consideration could be confirmed by looking at the shape of the first three eigenvectors. In Fig. 6b the normalized eigenvectors corresponding to the three larger eigenvalues are shown. $U_1$ is a superposition of a damped high-frequency oscillation and a low-frequency oscillation, appearing as a modulation. $U_2$ and $U_3$ have the same general behavior as $U_1$, save the different damping and phase. $U_2$ is approximately in anti-phase with $U_1$, $U_3$ is approximately in quadrature with $U_1$.

The barycentric spectrum of the LD group is shown in Fig. 6c. As in the previous case (Fig. 4c), the trace is obtained by summing the first three eigenvectors with the weights related to the barycenter of the polyhedron containing the LD group, see the red cross in Fig. 6a. In the figure, the contributing weighted eigenvectors are shown. The first eigenvector essentially coincides with the barycentric trace, the contribution of $U_2$, and $U_3$ being small. Similar behavior is found in the barycentric trace of the HDnp group, Fig. 6d. Instead, the HDp group is characterized by a marked influence of $U_2$ and $U_3$ eigenvectors, that differentiate its trace from those of the other groups, see Fig. 6e. To rationalize these findings, we note that it has been demonstrated that densely polymer-tethered colloidal particles, studied by Brillouin light scattering, have a modified acoustic response compared to uniform particle-in-polymer systems^56^. Following this suggestion, we note that when the cross-linking polymers in the high-density nanospheres are cut by the plasma action, these become acoustically similar to the low-density nanospheres, where inter-particle polymer cross-linking is absent. Our analysis shows that the acoustic response depends not on particle surface density, but on the presence or absence of polymer cross-linking between gold nanospheres, a fact that opens a new dimension in the control of nanoparticle acoustics response in complex environments.

## Conclusions

We have developed and detailed a protocol to analyze time-resolved spectra. Using a hierarchical clustering algorithm, it is possible to identify data outliers in a simple and meaningful way. Outlier removal allows us to purify spectra from the influence of errors that do not stem from the limitations of instruments but have a singular origin and are non-reproducible. As non-exhaustive examples, consider mislabeling of data by researchers, unintended measurements on different features of a sample (e.g. contaminants), unintended alteration of the instruments’ parameters. The analysis of the selected experimental traces with the SVD, in addition to confirming the subdivision into groups found by the hierarchical clustering, allows to reduce data dimensionality and focus on a small number of eigenvectors to reconstruct data.

The proposed analysis allows us to extract useful information from data that have been collected under challenging experimental conditions. The gold nanoparticles investigated in this work have been measured with an off-resonance mid-infrared pump instead of a pump near the plasmon absorption peak, as usual in the literature. As a consequence, the energy delivery to the nanoparticle is smaller by orders of magnitude compared to an in-resonance pump (see the SI, section 4 for an estimate of the absorption cross-section).

Although the wavelength choice for the pump beam ($\lambda _{pu}=1560$ nm) is not optimized for measurement sensitivity, the use of short-wavelength infrared beams is interesting to explore a spectral region that is not usually probed in time-resolved experiments. An infrared pump is an attractive choice to investigate biological materials because is known as non-carcinogenic. As an example of a potential implementation, consider both the optical absorption coefficient and the scattering coefficient of the human subcutaneous adipose tissue, that are reduced in the near-infrared/short-wavelength infrared region ($0.7 \upmu m<\lambda <1.6 \upmu m$)^57^, implying an increase in light penetration depth and reduced scattering. It has been found that a mild hyperthermia treatment (with a temperature increase of less than $5\, ^{\circ }\hbox {C}$) causes pro-apoptotic responses in particle-labeled cancer cells, so that even a wavelength not optimized for absorption may be useful in cancer thermal treatment^58,59^.

The near-infrared probe beam at 780 nm is in a spectral window where both water and hemoglobin have low absorption, being thus highly penetrating in living tissues (e.g. brain tissue)^60^. Both wavelength have a reduced scattering in tissues compared to visible wavelengths, thus enhancing the contrast in sensing applications.

In all these cases, the application of time-resolved measurement techniques should benefit from the data analysis presented in this work.

Finally, we would like to highlight that the procedures outlined in this work can be applied in different experimental conditions and are thus general. Consider an experiment whose output depends on a set of control parameters (e.g., density, temperature, pH, concentration, etc.) that are under the experimenters’ control. Repeating the experiment varying the parameters in discrete steps produces different groups labeled by different control parameters. In this case, the analysis presented here should help to extract differences among these groups by looking at the eigenvectors and to highlight if dominant features common to all groups are present. The detailed interpretation of the data is specific to the experimental situation. It needs some clues coming from what is known from the physics, chemistry, biology of the problem at hand and possibly an interpretative model to be tested.

## Supplementary information

Supplementary material 1
