# Supplementary material for: Optical and mechanical properties of streptavidin-conjugated gold nanospheres through data mining techniques
Source: Sci Rep. 2020 Oct 1;10:16230. doi: 10.1038/s41598-020-72534-1 (PMC7530730; doi:10.1038/s41598-020-72534-1)
Supplement: Supplementary file 1 — Supplementary material 1 [file 41598_2020_72534_MOESM1_ESM.pdf]

Supplementary Information for “Optical and Mechanical properties of streptavidin-conjugated gold nanospheres through data mining techniques”

Simone Peli[1,2], Andrea Ronchi[1,2,3], Giada Bianchetti[1,2,+], Francesco Rossella[4], Claudio Giannetti[1,2], Marcella Chiari[5], Pasqualantonio Pingue[4], Francesco Banfi[1,6], and Gabriele Ferrini*[1,2]

[1]Interdisciplinary Laboratories for Advanced Materials Physics (I-LAMP), Università Cattolica del Sacro Cuore, I-25121 Brescia, Italy
[2]Dipartimento di Matematica e Fisica, Università Cattolica del Sacro Cuore, I-25121 Brescia, Italy
[3]Department of Physics and Astronomy, KU Leuven, Celestijnenlaan 200D, 3001 Leuven, Belgium
[4]NEST, Scuola Normale Superiore and CNR – Istituto Nanoscienze, piazza San Silvestro 12, I-56127, Italy
[5]Istituto di Chimica del Riconoscimento Molecolare, CNR, Milano, Italy
[6]FemtoNanoOptics Group, Université de Lyon, CNRS, Université Claude Bernard Lyon 1, Institut Lumière Matière, F-69622 Villeurbanne, France
[+]now at Fondazione Policlinico Universitario A. Gemelli IRCSS, Rome, Italy and Dipartimento di Neuroscienze, Università Cattolica del Sacro Cuore, Rome, Italy
[*]gabriele.ferrini@unicatt.it

ABSTRACT

This is a supplementary information document to complement the findings in the main text of the article: “Optical and Mechanical properties of streptavidin-conjugated gold nanospheres through data mining technique”. Here, we explain the sample treatments, the imaging techniques, and show some theoretical estimations of interest for the laser pulse-nanosphere interaction.

1 Plasma treatment

The high density samples have been subject to an Oxygen plasma treatment using a low-pressure plasma system model PICO by Diener Electronic, 50 W for 90 s, to partially remove the polymer coating and expose the bare nanospheres.

2 Sample imaging

The sample imaging has been carried out with Atomic Force Microscopy (AFM) and Scanning Electron Microscopy (SEM). In the following, we briefly highlight the techniques and discuss the measurement results.

The topographic AFM measurements were performed employing a commercial Dimension Icon AFM (Bruker) operating in ”peak force tapping mode” (PFT)[1] and using ”ScanAsyst-air” probes (nominal elastic constant 0.4 N/m). PFT-mode imaging is performed by nanoindenting the sample pixel-by-pixel, controlling the force applied by the tip. This imaging mode allowed us to avoid any NPs scrubbing induced by the AFM probe. The results of a typical AFM topographical scanning on a sample without plasma treatment is shown in Fig. 1. The average height of the nanospheres is compatible, within errors, with the nominal diameter of 40 nm of the streptavidin-conjugated gold nanospheres declared by the vendor.

The SEM imaging exploited the charge compensator apparatus of our system (model Merlin, 30 keV FEG system, ZEISS) to perform direct imaging on NPs on sapphire, avoiding the need for any conductive coating layer on the sample surface. This methodology allowed high-resolution imaging on NPs, without topographic artifacts induced by the conductive film. The SEM images are shown in Fig. 1 b)-c) in the main text.

3 Surface density estimates

The surface density estimation of the samples on which the time-resolved optical measurements were taken has been carried out using AFM topographic images. This allowed the control of the height of the single nanosphere or cluster of nanospheres, to be sure we were imaging the nominal 40 nm diameter gold nanospheres. We found a height of 40 ± 7 nm (with background

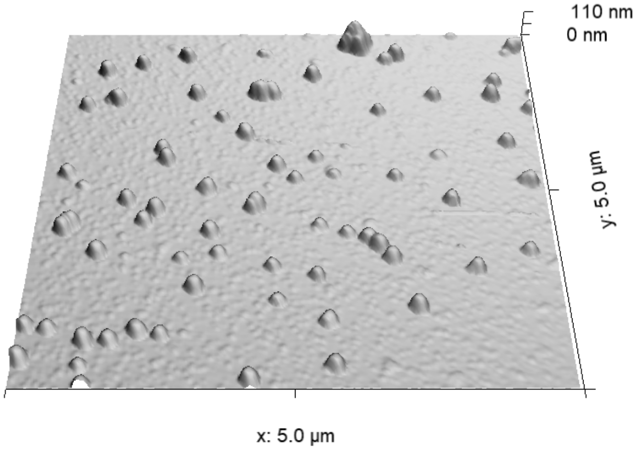

Figure 1. The topography of the gold nanospheres adhered to a sapphire surface by peak force tapping mode AFM.

subtraction) on 11 random samples from high density and low-density samples. The count of the surface density of the nanoparticle clusters has been conducted on AFM image topography using ImageJ[2] (typical scanning area of 5-20 μm^2, depending on the sample). On low-density samples, we found an average area of 3500 ± 900 nm^2, corresponding to an average radius of 30 ± 4 nm. On the high-density samples, we found an average area of 9000 ± 6000 nm^2, corresponding to an average radius of 55 ± 15 nm. We have to take into account that on the streptavidin-conjugated spheres a corona of polymer/antibody molecules contributes to a bigger AFM topographic cross-section and that the convolution with the tip radius of curvature (typically 20 nm) tends to increase the apparent dimensions of small particles in the images. Considering that the nominal core radius of the gold nanospheres is 20 nm, the results found for the low-density sample are compatible with clusters composed on average of a single nanosphere. Instead, the high-density sample the average cluster area is compatible with clusters formed on average by 3 ± 2 nanospheres. The SEM images have been used to verify the long-range uniformity of the surface distributions of nanoparticles. In the SEM images, a halo can be seen around each nanoparticle, that we attribute to a different electron scattering from the polymer around each particle (see Fig. 1 in the main text).

4 Absorption cross-section

The extinction cross-section for R= 20 nm radius gold spheres in a polymer matrix has been evaluated using the standard expression, keeping only the dipolar terms in Mie's theory[3,4]. In this approximation, the absorption cross-section is valid only for small particles (2R $\ll \lambda/10$) and the extinction is dominated by absorption.

The composite material absorption cross section in this approximation is written:

$$\sigma_{abs} = \frac{9V\omega}{c}\varepsilon_m^{3/2}\frac{\varepsilon_2}{(\varepsilon_1 + 2\varepsilon_m)^2 + \varepsilon_2^2}$$

where $\varepsilon_m = n_m^2 = 1.36^2$ is the polymer dielectric constant where the sphere is supposed to be immersed and will be assumed frequency-independent and real. The polymer refraction index was obtained with an AnaLight refractometer. The Johnson and Christy refractive indexes for gold[5] have been used to retrieve the spectral dependence of ε_1 and ε_2. In Fig. 2, the absorption cross-section as a function of wavelength is reported. Note that at the pump wavelength, marked by a red dot, the cross-section is three orders of magnitude less than in resonance.

5 Electron and lattice temperature estimations

The gold nanospheres have an average radius of R=20 nm. The refraction index of the hosting medium is $n_m = 1.36$. The pump laser emits sub-ps pulses at a repetition rate of 100 MHz with an average power of 2 mW, resulting in an energy of 20 pJ/pulse. The pump wavelength is $\lambda_{pu} = 1560$ nm, with a single particle pump absorption cross section $\sigma_{pu} = 2.7$nm^2 (see the preceding section). The impinging pump beam is focalized through a lens to a spot size of area A_{pu} in the focus of the lens. With an objective of numerical aperture NA=0.55 the spot radius in the focus is $0.61\lambda_{pu}/NA = 1.77\mu$m, the spot area is $A_{pu} = 9.4\mu$m^2.

We define I_{pu}^0 as the pump intensity measured at a detector after the sample without the nanosphere in the focus of the impinging beam, I_{pu}^1 is the intensity at the detector *with* the nanosphere in the focus of the impinging beam. Using the optical

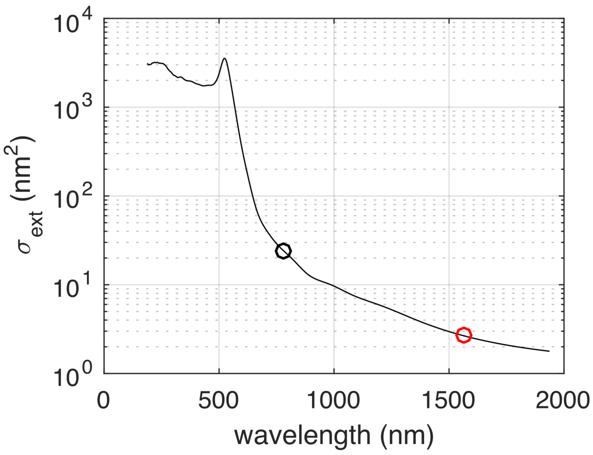

Figure 2. Absorption cross-section for a 20 nm radius gold nanosphere as a function of wavelength. The red dot mark the pump wavelength, the black dot mark the probe wavelength.

theorem[6,7] $\frac{I^0_{pu}-I^1_{pu}}{I^0_{pu}} = \Delta I_{pu}/I^0_{pu} = \sigma_{pu}/A_{pu}$. Since the time width of the pulse and spot area are the same, the relation is valid also for the variation in the energy of a single pulse, $u^0_{pu} - u^1_{pu} = u_{pu} = \sigma_{pu} u^0_{pu}/A_{pu}$, where u^0_{pu} is the impinging pulse energy without the nanosphere, u^1_{pu} is the energy of the pulse after passing through the nanosphere, u_{pu} is the absorbed energy in a single nanosphere. With the values shown above the absorbed energy per nanosphere is $u_{pu} = 45\ h\nu_{pu}$. The maximum electronic temperature, neglecting thermal transport and energy exchange with the lattice, can be estimated from the energy balance between the rate of optical energy density input (P_{pu}/V), where P_{pu} is the absorbed pump power by the nanoparticle electron gas and V the nanoparticle volume, and the energy needed to increase the electronic temperature ($C_e \frac{dT_e}{dt}$), where C_e is the electronic thermal capacitance and T_e is the electronic temperature. Taking into account that the electronic thermal capacitance is proportional to temperature $C_e = \gamma T_e$, $\gamma = 100$ J/(m^{3}K^2) for gold.

$$C_e \frac{dT_e}{dt} = \gamma T_e \frac{dT_e}{dt} = \frac{\gamma}{2} \frac{dT_e^2}{dt} = \frac{P_{pu}}{V}$$

Integrating on the pulse duration we obtain for the peak electronic temperature variation $\Delta T_e(max) = T_e(max) - T_e(0) = \sqrt{T_e^2(0) + 2\frac{u_{pu}}{\gamma V}} - T_e(0) \approx 6$ K. The maximum lattice temperature could be estimated by supposing that the energy density stored in the electrons is finally released to the lattice with thermal capacity $C_l = 2.46 \times 10^6$ J/(m^{3}K), resulting in a peak lattice temperature variation $\Delta T_l(max) = u_{pu}/(VC_l) \approx 0.07$ K.

The variation of the nanosphere radius due to the temperature increase is $\Delta R = R(1 - \alpha \Delta T_l(max)) \approx 0.02$ pm, where $\alpha = 14 \times 10^{-6}$ is the linear expansion coefficient for gold.

6 Probe transmission and pump induced cross-section variation

The probe laser emits sub-ps pulses at a repetition rate of 100 MHz with an average power of 0.25 mW, resulting in an energy per pulse of 2.5 pJ/pulse. The probe wavelength is $\lambda_{pr} = 780$ nm, with a single particle probe absorption cross section $\sigma_{pr} = 25$nm^2 (see the preceding section). With a lens of numerical aperture NA=0.55 the spot radius in the focus is $0.61\lambda_{pr}/NA = 0.87\mu$m, and the area is $A_{pu} = 2.35\mu$m^2. The presence of the nanosphere gives a probe transmission variation estimated as[6,7] $\frac{I^0_{pr}-I^1_{pr}}{I^0_{pr}} = \Delta I_{pr}/I^0_{pr} = \sigma_{pr}/A_{pr} = 1 \times 10^{-5}$. This would be the transmission variation measured in standard transmission microscopy measurements. However pump-probe experiments monitor the change in the probe beam intensity induced by the pump beam, i.e. $\Delta(\Delta I_{pr})/I^0_{pr} = \Delta\sigma_{pr}/A_{pr}$. From experiments we measure a peak relative transmission variation of the order of $\Delta T/T = -2/-5 \times 10^{-5}$, which is of the same order of magnitude of the absolute transmission variation estimated above, taking into account the sign that comes from the definition of transient transmission (see main text). Using the measured average transmission variation in the pump-probe experiment and the above relation, the estimated peak variation in cross-section due to the pump excitation is $\Delta\sigma_{pr} \approx 80$ nm^2. The pump induces a cross-section peak variation much higher than the estimated particle cross-section for a static transmission experiment.

7 Clustering algorithm

The general workflow for a clustering algorithm is as follows. Given n experimental traces x_i, consider initially n singleton clusters $\omega_i = x_i$. Then, determine the two nearest clusters ω_i and ω_j using an appropriate distance (or linkage) $d(\omega_i, \omega_j)$. Merge ω_i and ω_j: $\omega_{ij} = \{\omega_i, \omega_j\}$, therefore obtaining a solution with $n-1$ clusters. Repeat the process until a single cluster is obtained. In the Ward's method implementation used in this work[8], the squared distance $d(\omega_i, \omega_j)^2$ is defined as the *increase* of the squared within-cluster distances (defined as the sum of the squares of the distances between all objects in the cluster and the centroid of the cluster) when the cluster merges and is computed as:

$$d(\omega_i,\omega_j)^2 = 2\Big(\sum_{x\in\omega_i\cup\omega_j} ||x-m_{\omega_i\cup\omega_j}||^2 - \sum_{x\in\omega_i} ||x-m_{\omega_i}||^2 - \sum_{x\in\omega_j} ||x-m_{\omega_j}||^2\Big) = \frac{2n_in_j}{n_i+n_j}||m_{\omega_i}-m_{\omega_j}||^2 \tag{1}$$

where m is the centroid of the cluster. The metric $||...||$ used to calculate the distance is the Euclidean distance between experimental traces. The factor of 2 is used so that the distance between two singleton clusters is the same as the Euclidean distance. At each step, the two clusters that merge are the ones that contribute to the smallest increase of the overall sum of the squared within-cluster distances.

8 Mechanical analysis

We show in Fig. 3 the full set of fitting parameters found in the mechanical analysis with relative errors. Refer to the corresponding section in the main text. Here we notice a few points that have values and associated errors considerably larger than the others, so that including them in the graph prevents a correct overall visualization. This is why only a subset of these points is reported in Fig. 5 b), c), d), e) in the main text. We stress that a large error associated with one parameter does not imply that the other parameters, associated with the same fit, are affected by large errors (see, e.g., points 9, 16).

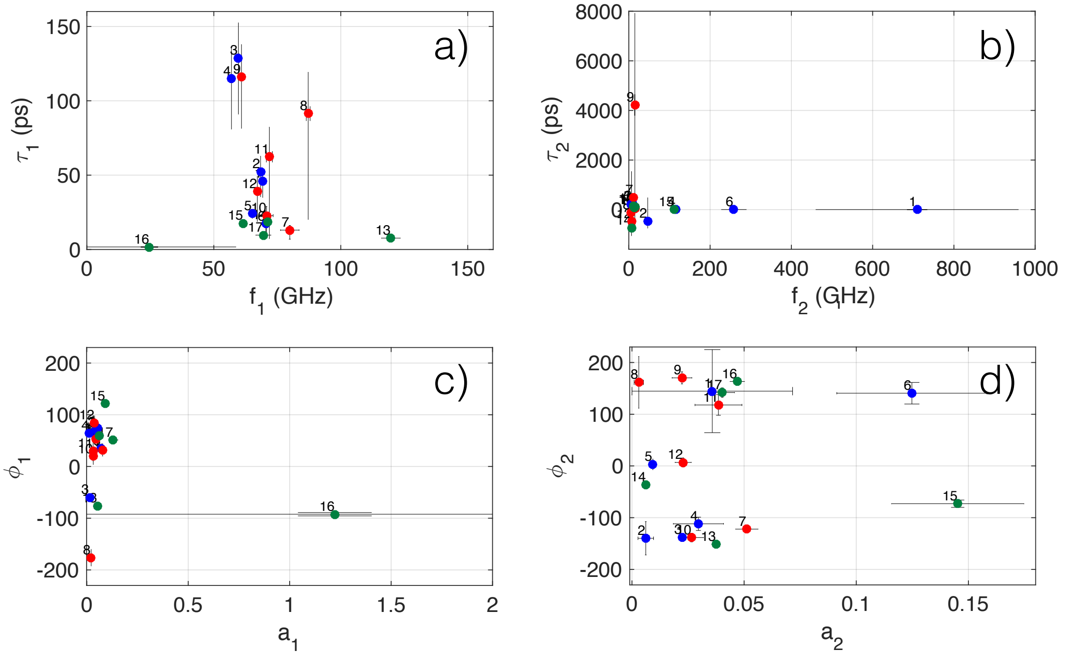

Figure 3. The complete set of fitting parameters for the mechanical analysis are shown. a) and b) reports the oscillation frequencies versus associated exponential damping times for the first (f_1 vs τ_1) and second (f_2 vs τ_2) oscillators. c) and d) reports the amplitude versus phase for the first (a_1 vs ϕ_1) and the second (a_2 vs ϕ_2) oscillators.

Acknowledgments

G.F. acknowledges financial support from the MIUR - PRIN 2017 grant in the frame of the aSTAR Project (2017RKWTMY_003). F.R. acknowledges the financial support by MIUR - PRIN 2017 Project PELM, protocol n. 20177PSCKT.2. F.B., S.P., and F.R. acknowledge financial support from the MIUR - Futuro in Ricerca 2013 Grant in the frame of the ULTRANANO Project. F.B. acknowledges financial support from the IDEXLYON project Programme Investissements d'Avenir (ANR-16-IDEX-0005). F.B. and S.P. acknowledge financial support from fondazione E.U.L.O. This work has been partially supported by the Università Cattolica del Sacro Cuore through D.3.1 and D.2.2 grants.

Author Contributions

G.F. conceived the project and developed the data analysis. F.B., C.G., and G.F. supervised and organized the research. S.P., A.R, G.B. made the optical time-resolved measurements and fitted the data. M.C. made the samples and characterized their chemistry P.P. made the SEM and AFM analysis and plasma-treated the samples. F.R. made the electron lithography for the samples substrate. G.F. wrote the manuscript with major inputs from F.B. All authors discussed the results and revised the manuscript.

References

1. Pittenger, B., Erina, N. & Su, C. Quantitative mechanical property mapping at the nanoscale with peakforce qnm. *Appl. Note Veeco Instruments Inc* 1–12 (2010).

2. Rasband, W. S. *et al.* Imagej (1997). URL https://imagej.nih.gov/ij/index.html.

3. Voisin, C., Del Fatti, N., Christofilos, D. & Vallée, F. Ultrafast electron dynamics and optical nonlinearities in metal nanoparticles. *J. Phys. Chem. B* **105**, 2264 (2001).

4. Hartland, G. V. Optical studies of dynamics in noble metal nanostructures. *Chem. Rev.* **111**, 3858–3887 (2011).

5. Johnson, P. B. & Christy, R.-W. Optical constants of the noble metals. *Phys. review B* **6**, 4370 (1972).

6. Li, Z., Aleshire, K., Kuno, M. & Hartland, G. V. Super-resolution far-field infrared imaging by photothermal heterodyne imaging. *The J. Phys. Chem. B* **121**, 8838–8846 (2017).

7. Crut, A., Maioli, P., Del Fatti, N. & Vallée, F. Optical absorption and scattering spectroscopies of single nano-objects. *Chem. Soc. Rev.* **43**, 3921–3956 (2014).

8. Kaufman, L. & Rousseeuw, P. J. *Finding groups in data: an introduction to cluster analysis* (John Wiley & Sons, 2009).
